# Multiple sevoflurane exposures during mid‐trimester induce neurotoxicity in the developing brain initiated by 15LO2‐Mediated ferroptosis

**DOI:** 10.1111/cns.14236

**Published:** 2023-06-07

**Authors:** Qian Jiang, Cong Wang, Qiushi Gao, Ziyi Wu, Ping Zhao

**Affiliations:** ^1^ Department of Anesthesiology Shengjing Hospital of China Medical University Shenyang China

**Keywords:** 15‐lipoxygenase 2, ATM, ferroptosis, neurotoxicity, sevoflurane

## Abstract

**Aims:**

Mid‐gestational sevoflurane exposure may induce notable long‐term neurocognitive impairment in offspring. This study was designed to investigate the role and potential mechanism of ferroptosis in developmental neurotoxicity induced by sevoflurane in the second trimester.

**Methods:**

Pregnant rats on day 13 of gestation (G13) were treated with or without 3.0% sevoflurane, Ferrostatin‐1 (Fer‐1), PD146176, or Ku55933 on three consecutive days. Mitochondrial morphology, ferroptosis‐relative proteins, malondialdehyde (MDA) levels, total iron content, and glutathione peroxidase 4 (GPX4) activities were measured. Hippocampal neuronal development in offspring was also examined. Subsequently, 15‐lipoxygenase 2 (15LO2)‐phosphatidylethanolamine binding protein 1 (PEBP1) interaction and expression of Ataxia telangiectasia mutated (ATM) and its downstream proteins were also detected. Furthermore, Morris water maze (MWM) and Nissl's staining were applied to estimate the long‐term neurotoxic effects of sevoflurane.

**Results:**

Ferroptosis mitochondria were observed after maternal sevoflurane exposures. Sevoflurane elevated MDA and iron levels while inhibiting GPX4 activity, and resultant long‐term learning and memory dysfunction, which were alleviated by Fer‐1, PD146176, and Ku55933. Sevoflurane could enhance 15LO2‐PEBP1 interaction and activate ATM and its downstream P53/SAT1 pathway, which might be attributed to excessive p‐ATM nuclear translocation.

**Conclusion:**

This study proposes that 15LO2‐mediated ferroptosis might contribute to neurotoxicity induced by maternal sevoflurane anesthesia during the mid‐trimester in the offspring and its mechanism may be ascribed to hyperactivation of ATM and enhancement of 15LO2‐PEBP1 interaction, indicating a potential therapeutic target for ameliorating sevoflurane‐induced neurotoxicity.

## Graphical abstract

Ferroptosis contributes to the neurotoxicity induced by sevoflurane anesthesia during mid‐trimester in the offspring. Sevoflurane could enhance 15LO2‐PEBP1 interaction and activate ATM and its downstream P53/SAT1 signaling which might be attributed to excessive p‐ATM nuclear translocation.

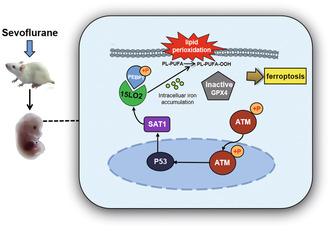

## 1 INTRODUCTION

Fetal surgery has become an acceptable option for certain neonatal ailments owing to advancements in prenatal diagnosis.^1^ Fetuses with severe abnormalities may require open mid‐gestation operations, which demand varying degrees of analgesia, sedation, or anesthesia for both mother and fetus to optimize surgical conditions and obtain beneficial outcomes.^2^ However, mid‐gestational anesthetics exposure has been demonstrated to induce prolonged neurocognitive deficits in offspring, suggesting the potential neurotoxicity of anesthetics on developing brain.^3^, ^4^, ^5^, ^6^ Sevoflurane is one of the most widely used general anesthetics and is currently favored for general anesthesia during pregnancy, which requires high doses of anesthetics to avoid intraoperative uterine contractions.^7^, ^8^ Our previous studies have proved that repeated sevoflurane exposures during mid‐trimester could cause long‐term learning and memory dysfunction in the offspring.^4^, ^9^ To date, its mechanism remains unclear.

Ferroptosis, a novel type of programmed cell death, can be triggered by inhibition of cystine uptake, inactivation of GPX4, iron overload, and subsequent accumulation of lipid ROS^10^, ^11^ and can be effectively rescued by specific inhibitors such as Fer‐1.^11^ GPX4, Solute carrier family 7 member 11 (SLC7A11) and Acyl‐CoA synthetase long‐chain family member 4 (ACSL4) are considered key modulators of ferroptosis.^12^, ^13^ Emerging evidence indicates that ferroptosis as well as lipid peroxidation are intimately correlated with neurodegenerative diseases,^14^, ^15^, ^16^, ^17^ tumors,^18^, ^19^, ^20^ and ischemia‐reperfusion injury processes.^21^, ^22^, ^23^, ^24^, ^25^ Given that the developing brain has a higher content of polyunsaturated fatty acids (PUFAs) in cell membranes but is more vulnerable endogenous antioxidant defense mechanisms compared to the mature brain, the fetal or preterm brain is more sensitive to lipid peroxidation and oxidative stress‐induced cell death than the full‐term and adult brain.^26^, ^27^ Taken together, the developing brain is more susceptible to lipid peroxidation‐induced ferroptosis. Furthermore, previous studies confirmed that isoflurane exposure could trigger ferroptosis, which induced developmental neurotoxicity.^28^, ^29^ Nevertheless, there is insufficient evidence to prove the attribution of ferroptosis to sevoflurane‐induced neurotoxicity.

Ferroptosis can be driven by lipid peroxides catalyzed by lipoxygenase (LOX).^30^ 15LOX, especially arachidonate 15‐lipoxygenase (ALOX15) is a crucial ferroptosis‐related enzyme, which can modulate cellular resistance to ferroptosis.^31^ As a subtype of 15LOX, 15LO2 is highly expressed in the cerebral cortex, hippocampus, and olfactory bulb.^32^ PEBP1, a specific 12,15‐lipoxygenase regulator, forms a complex with 15LOX which could target PUFAs on cell membranes to oxidize free PUFAs to PUFA‐PEs.^33^, ^34^, ^35^ In the presence of GPX4 deficiency or inactivation, lipid peroxides accumulate and thus trigger ferroptosis.^36^ The enzymatic reaction catalyzed by 15LOX/PEBP1 is thought to dominate the initiation phase of lipid peroxidation in ferroptosis.^37^

As an important regulator of the development of the central nervous system,^38^ ATM also functions as an essential kinase of ferroptosis.^39^ There is a complex interaction between ATM and P53 in ferroptosis.^40^, ^41^ P53 may modulate ferroptosis by either transcriptional or post‐translational mechanisms in ferroptosis.^42^, ^43^ P53 mediates the activation of spermidine/spermine N 1‐acetyltransferase 1 (SAT1) to trigger ferroptosis in response to reactive oxygen species (ROS)‐induced stress under GPX4 inactivation.^42^, ^44^ SAT1‐induced ferroptosis is involved in neurodegenerative disease^45^ and traumatic brain injury,^46^ indicating that SAT1 might participate in ferroptosis‐related neurotoxicity induced by anesthetics. Notably, ALOX15 has been established as a downstream effector of P53/SAT1 signaling.^44^

Hence, this study aimed to elucidate whether ferroptosis contributes to sevoflurane‐induced neurotoxicity in offspring during the mid‐trimester and its potential mechanism. We hypothesized that 15‐mediated ferroptosis onset in the fetal brain after maternal multiple 3.0% sevoflurane exposures and consequently induced neurotoxicity in the offspring. Enhancement of 15LO2‐PEBP1 and activation of ATM and P53/SAT1 signaling potentially contributed to sevoflurane‐induced ferroptosis.

## 2 MATERIALS AND METHODS

### 2.1 Animals and housing

The research protocol was approved by the Ethics Committee of Shengjing Hospital, China Medical University (No. 2019PS198K). Every effort was made to minimize animal suffering and the number of animals. Specific Pathogen‐Free (SPF) Sprague–Dawley (SD) rats, weighing 190–240 g for females and 260–320 g for males, were housed at 23 ± 1°C with a regular light/dark cycle and free access to water and food. A vaginal smear procedure was performed on the female to determine the time of conception. If sperm can be seen in the smear under the microscope, the female rat is marked G0. The offspring rats were labeled P0 on the day of birth.

### 2.2 Experimental design and sevoflurane exposure

**FIGURE 1:**
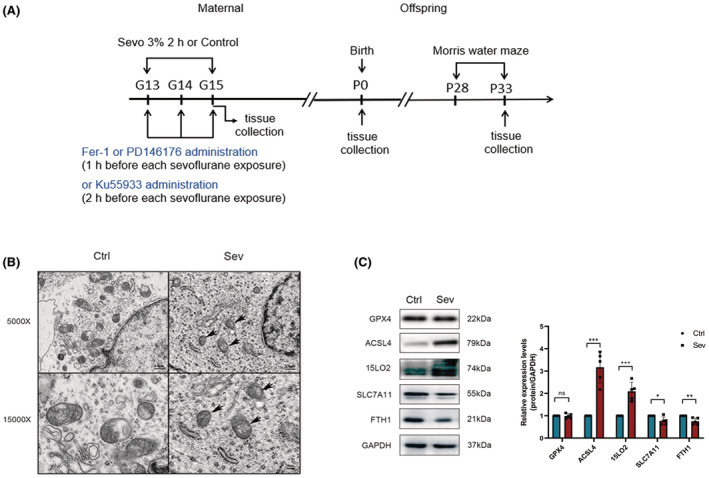
(A) Flow diagram of the study protocol. (B) TEM revealed subcellular structural alterations of neurocytes in fetal rat brain after sevoflurane exposures. Photographs at 5000× and 15,000× under TEM with scales of 2.5 and 1 μm, respectively (*n* = 3 per group). The black arrows show the characteristic mitochondria of ferroptosis. (C) Western blot images and quantification analysis of GPX4, ACSL4, 15LO2, SLC7A11, and FTH1 in the fetal rat brain (*n* = 5 per group). Values are presented as mean ± SD. *p < 0.05, **p < 0.01, and ***p < 0.001 vs. the Ctrl group; 15LO2, 15‐lipoxygenase 2; ACSL4, Acyl‐CoA synthetase long‐chain family member 4; FTH1, ferritin heavy chain; GPX4, glutathione peroxidase 4; ns., no significance; SLC7A11, Solute carrier family 7 member 11; TEM, transmission electron microscope.

A flowchart of the study protocol is shown in Figure 1A. Pregnant rats were placed in a dedicated plastic chamber with ambient gas at a flow rate of 2 L/min. Concentrations of carbon dioxide, oxygen, and anesthetics were continuously measured by a calibrated lateral flow analyzer. G13 rats were exposed to a nitrogen‐oxygen mixture of 30% oxygen or a 3.0% sevoflurane‐30% oxygen gas mixture for 2 h on 3 consecutive days. Any pregnant rat was excluded from the study if it developed any of the following conditions while under anesthesia: cyanosis, apnea, and death.

### 2.3 Drugs administration

Fer‐1 solubilized in saline and 1% dimethyl sulfoxide (DMSO) and PD146176 (a specific 15LOX inhibitor) dissolved in corn oil containing 1% DMSO were administered intraperitoneally to rats at a dose of 5 mg/kg 1 h before each exposure, respectively. Similarly, 0.5 mg/kg Ku55933 (an ATM inhibitor), which is diluted in saline containing 1% DMSO, was intraperitoneally administered 2 h previously. Afterward, the rats were relocated to the original cages and randomly divided as follows: control (Ctrl) group, sevoflurane (Sevo) group, sevoflurane+Fer‐1 (Sevo+F) group, control+Fer‐1 (Ctrl+F) group, sevoflurane+PD146176 (Sevo+P) group, control+PD146176 (Ctrl+P) group, sevoflurane+Ku55933 (Sevo+K) group, and control+Ku55933 (Ctrl+K) group.

### 2.4 Tissue preparation and sectioning

As 10 pregnant rats were randomly assigned to each group, fetuses were removed from pregnant rats (5 in each group) by cesarean section at 12 h after gas exposure on G15, and the whole fetal brain tissue was harvested immediately. The remaining pregnant rats (5 in each group) were assigned to labor naturally. On P0, the whole brain and hippocampus of the offspring were extracted, respectively. MWM tests were performed from P28 to P33. To avoid the impact of gender differences on the experiment, we randomly selected offspring for subsequent experiments at P0 and P28 in the offspring of each pregnant rat in a male‐female sex ratio of 1:1. After all behavioral tests were completed, we collected P33 brain tissue to measure neuronal density in the hippocampus.

### 2.5 Transmission electron microscope

The hippocampal rudiment of the fetal brain was taken under a dissecting microscope, and tissue preparation was performed by the transmission electron microscopy slice preparation kit (GMS11007, GENMED Corporation). The specimens were segmented at a thickness of 70 nm, followed by double staining with 3.0% uranium acetate‐lead citrate. After the sections were prepared, transmission electron microscopy was applied to observe and photograph the sections.

### 2.6 Western blotting analysis

The tissue was homogenized in a radioimmunoprecipitation assay lysis buffer (P0013B, Beyotime Biotechnology) with a 1.0% protease inhibitor cocktail (P8349, Sigma‐Aldrich) or by the Nuclear and Cytoplasmic and Protein Extraction Kit (P0028, Beyotime) to extract cytoplasmic‐nucleoprotein, and then incubated on ice for 30 min. Protein concentrations were determined using a BCA Kit (P0010, Beyotime). We used 6% or 10% SDS‐polyacrylamide gels for electrophoresis and then electrotransferred the proteins to polyvinylidene difluoride membranes (IPVH0010, Millipore). The PVDF membranes were blocked with 5% skimmed milk for 2 h, followed by incubation with primary antibodies: ACSL4 (1:2000, ab155282, Abcam), GPX4 (1:4000, ab125066, Abcam), PTGS2 (1:600, 66351‐1‐Ig, Proteintech Biotechnology), SLC7A11 (1:1000, ab175186, Abcam), FTH1 (1:1000, #4393, Cell Signaling Technology), 15LO2 (1:250, sc‐376795, Santa Cruz), PEBP1 (1:1000, ab76582, Abcam), p‐PEBP1 (1:1000, ab75971, Abcam), p‐ATM (1:750, sc‐47739, Santa Cruz), ATM (1:3000, 67586‐1‐Ig, Proteintech), GAPDH (1:8000, 60014‐1‐Ig, Proteintech) and Histone‐H3 (1:1000, 17168‐1‐AP, Proteintech), respectively. Antibodies against NeuN (1:1000, #24307, CST) and GAPDH were also used in P0 hippocampal samples. Any protein band was measured by enhanced chemiluminescence and quantified using ImageJ software (NIH Image, Bethesda, USA).

### 2.7 Immunofluorescence staining

Tissue was fixed in 4% paraformaldehyde (PFA) and then dehydrated in graded ethanol and embedded in paraffin. Brains were sliced into 3 μm thick coronal sections through the hippocampal rudiment. After heat‐mediated antigen retrieval with citrate buffer, sections were cultured with 10% fetal bovine serum for 30 min at room temperature and then incubated at 4°C with anti‐PTGS2 antibody (1:200, 66351‐1‐Ig, Proteintech), anti‐15LO2 antibody (1:200, sc‐376795, Santa Cruz) and anti‐PEBP1 antibody (1:200, ab76582, Santa Cruz) overnight, respectively. Subsequently, sections were incubated with specific Alexa Fluor 594 or 488 secondary antibodies (1:200, SA00013‐3, or SA00013‐2, Proteintech) for 4 h and DAPI (C1005, Beyotime) for 5 min at room temperature. Fluorescence images were collected using a Leica confocal fluorescence microscope (Leica TCS SP5 II), equipped with ZEN software. Negative controls were incubated with PBS without primary antibodies. Tissue was fixed in 4% PFA and then dehydrated in graded ethanol and embedded in paraffin.

### 2.8 MDA level assay

To assess lipid oxidation levels in fetal brains, MDA levels were measured by the Micro MDA Assay Kit (BC0025, Solarbio) according to the manufacturer's protocol. The absorbance of each sample was measured at 450, 532, and 600 nm, and the MDA content could be calculated according to the protocol.

### 2.9 Iron content assay

We applied the Tissue Iron Content Assay Kit (BC4355, Solarbio) to measure the iron content in fetal brains. We weighed 0.15 g of tissue in each tube, and 1.5 mL of extract was added and homogenized in an ice bath. According to the instructions, the absorbance at 520 nm was measured immediately by a multi‐mode microplate reader. Tissue iron content (μg/g) was calculated using the formula in the instruction manual.

### 2.10 GPX4 activity assay

The Micro Glutathione Peroxidase (GPX) Assay Kit (BC1195, Solarbio) was applied according to instructions. Five samples of 0.15 g from each group were prepared. A unit mass of sample catalyzing 1 nmol of GSH oxidation per minute in a reaction system was defined as a unit of enzyme activity, and glutathione peroxidase activity (U/g) was calculated according to the formula.

### 2.11 Immunohistochemical staining

Antigens reparation and paraffin segment dewaxing are identical to immunofluorescence staining. We chose the immunohistochemistry standard SP kit (SP‐9000, Zsbiotechnology). The sections were incubated overnight at 4°C with primary antibodies: anti‐NeuN (1:200, #24307, CST) or anti‐15LO2 (1:100, sc‐376795, Santa Cruz). The slides were then incubated with secondary antibodies, DAB, and hematoxylin. One to two random field of view were obtained on each slide under the 400× field of view of a Nikon C1 microscope. IOD values were analyzed by ImageJ.

### 2.12 Co‐Immunoprecipitation (Co‐IP)

The Pierce™ Magnetic Bead Method Direct IP/Co‐IP Kit (#88828, ThermoFisher) was selected and the procedure was performed according to instructions. Each tube weighed 0.1 g of fetal brain tissue and 150 μL of IP lysis/washing buffer was added to prepare tissue lysate, of which 50 μL was used as input samples, and 100 μL was used for Co‐IP. The primary antibodies used for Co‐IP were 15LO2 (sc‐376795, Santa Cruz) and PEBP1 (sc‐376925, Santa Cruz), respectively. Antibody dilutions at a final concentration of 8 μg/100 μL of primary antibodies for immunoprecipitation were prepared for each tube. Magnetic beads of 40 μL per tube were incubated with primary antibodies and tissue lysate for 1 h at room temperature on a DNA vertical mixer. After elution and neutralization, add the 5× electrophoresis loading buffer provided by the kit to the beads and then heat at 100°C for 5 min for protein denaturation. Discard the magnetic beads and collect the supernatant containing the target antigen for subsequent immunoblotting.

### 2.13 MWM test

The MWM test was applied as described^47^ to examine the spatial learning memory function of offspring. After the platform was removed, SPT was performed on P33 and the rats were allowed to swim independently for 120 s. The small animal behavior analysis system (Shanghai Mobile Data Co., Ltd.) was used for video acquisition and data processing throughout the experiment. After each trial, the rats were dried and heated for 5 min and then placed back in their original cages.

### 2.14 Nissl's staining

After paraffin embedding, we obtained coronal sections (2.5 μm thick) in the hippocampus and applied Nissl Staining Solution Kit (G1430, Solarbio) for staining. After the sections were dewaxed, the slices were first immersed in Cresyl Violet staining solution and incubated in a constant temperature incubator at 60°C for 1 h. After washing the slices with PBS for 5 min, the slices were placed under the microscope for differentiation. Images were collected from approximately the same area of the CA1 region under a 400× microscope, and relative mean optical density values were calculated by ImageJ software.

### 2.15 Statistical analysis

Statistical analysis was performed with SPSS 20.0 (SPSS Inc.) and GraphPad Prism 8.0.2 (GraphPad). Quantitative data are presented as mean ± standard error of the mean (SEM). For all continuous variables, the assumption of normality was tested using the Shapiro–Wilk test. If the assumption of normality was met, one‐way ANOVA followed by Tukey's post hoc test was used for multiple comparisons. Otherwise, the Kruskal–Wallis *H*‐test was used for comparison. Two‐way ANOVA was used to analyze escape latency data. GraphPad Prism 8.0.2 software was used for graphing. Statistical significance was defined as a *p* value < 0.05.

## 3 RESULTS

### 3.1 Maternal sevoflurane exposures induce ferroptosis in fetal rat brain

TEM was used to detect morphological changes in mitochondria to determine whether ferroptosis occurred in the fetal brain after maternal exposure to sevoflurane. The characteristic subcellular structure of ferroptosis^10^ was observed in the Sev group (Figure 1B). Both ACSL4 and 15LO2 were upregulated, while SLC7A11 and ferritin heavy chain (FTH1) were downregulated by sevoflurane as shown in Figure 1C. Nonetheless, no significant change in GPX4 expression was observed.

### 3.2 Ferrostatin‐1 attenuates the sevoflurane‐induced neurotoxicity of the offspring

**FIGURE 2:**
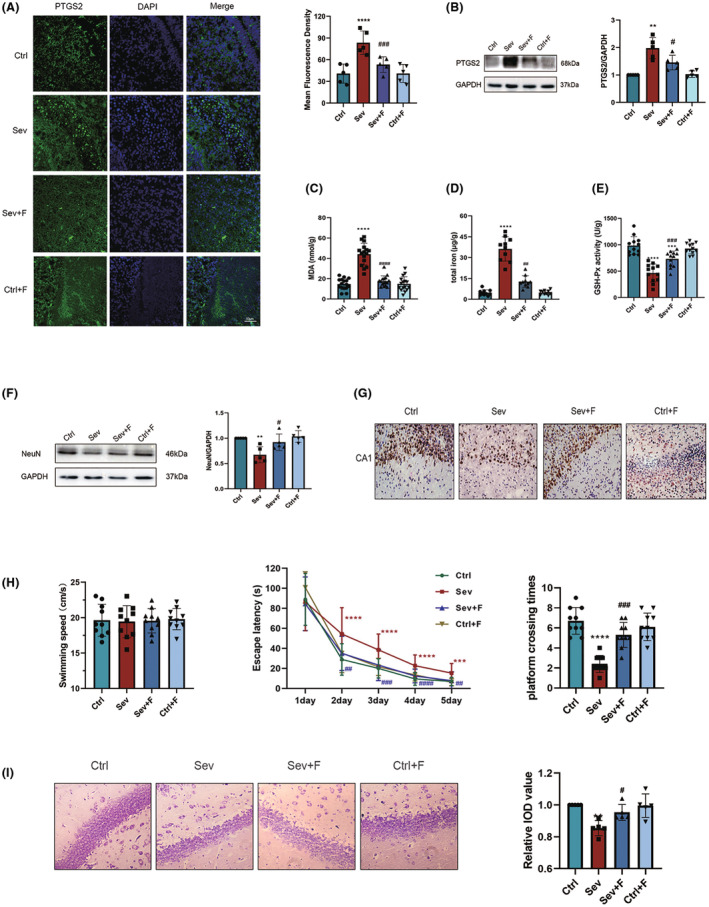
Effects of Fer‐1 on ferroptosis‐induced neurotoxicity in the offspring after maternal sevoflurane exposures. (A) Immunofluorescence images and quantitative analysis of PTGS2 (green) and DAPI (blue). *n* = 5 per group; Scale bar = 50 μm. (B) Western blot image and quantitative analysis of PTGS2 in the fetal brain (*n* = 5 per group). (C) MDA levels, (D) Iron content, and (E) GPX4 activities in the fetal rat brain (*n* = 5 per group). (F) Western blot image and quantitative analysis of NeuN in the hippocampus of the neonatal offspring (*n* = 5 per group). (G) Immunohistochemistry images of NeuN in P0 hippocampal CA1 region (*n* = 5 per group); Scale bar = 100 μm. (H) Results of MWM tests of the offspring from P28 to P33: swimming speed, escape latencies and platform crossing times (*n* = 10 per group). (I) Images and quantitative analysis of Nissl's staining in P33 offspring (*n* = 5 per group). Values are presented as mean ± SD. *p < 0.05, **p < 0.01, ***p < 0.001 and ****p < 0.0001 vs. the Ctrl group; ^#^ *p* < 0.05, ^##^ *p* < 0.01, ^###^ *p* < 0.001 and ^####^ *p* < 0.0001 vs. the Sev group. DAPI, 4′,6‐diamidino‐2‐phenylindole; Fer‐1, ferrostatin‐1; GPX4, glutathione peroxidase 4; MDA, malondialdehyde; MWM, Morris water maze; PTGS2, prostaglandin endoperoxide synthase 2.

Fer‐1 was administered to further clarify the contribution of ferroptosis in sevoflurane‐induced neurotoxicity. As a ferroptosis marker,^48^ prostaglandin‐endoperoxide synthase 2 (PTGS2) was widely expressed throughout the brain, and Fer‐1 could reduce elevated PTGS2 induced by sevoflurane (Figure 2A). The above results were also confirmed by Western blotting (Figure 2B). In addition, Fer‐1 decreased MDA and iron levels, revising GPX4 enzyme activity inhibition by sevoflurane (Figure 2C–E). Additionally, Fer‐1 diminished the NeuN expression in the hippocampus of neonatal dams (Figure 2F) and alleviated ferroptosis‐induced reduction of the neonatal hippocampus (Figure 2G). Moreover, similar to our previous studies,^9^ the MWM test showed no difference in spontaneous locomotor activity. Meanwhile, Fer‐1 shortened the sevoflurane‐prolonged latency periods and attenuated the decrease in platform crossing times after sevoflurane exposures (Figure 2H). In addition, Fer‐1 pretreatment may reduce neuronal density and alignment in the CA1 region of the hippocampus in offspring after maternal sevoflurane exposures (Figure 2I). Overall, these findings demonstrate that Fer‐1 might provide a protective effect against ferroptosis‐associated neurotoxicity induced by sevoflurane.

### 3.3 Sevoflurane enhanced 15LO2‐PEBP1 interaction

**FIGURE 3:**
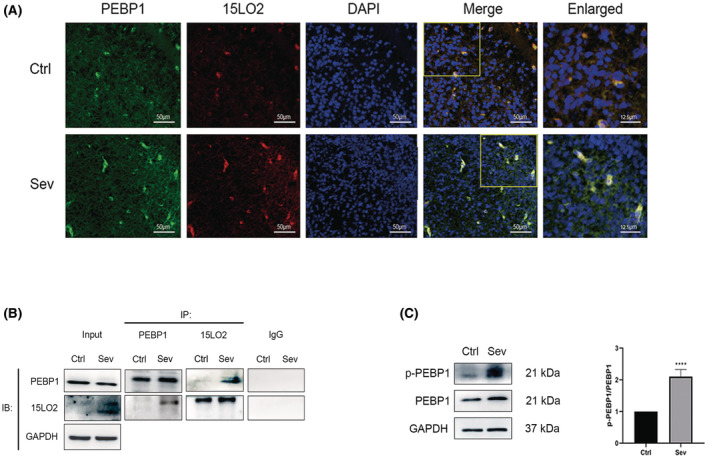
Maternal multiple sevoflurane exposures led to enhancement of the interaction of 15LO2‐PEBP1 in the fetal rat brain. (A) The immunofluorescence images of the fetal rat brain indicated the co‐localization of 15LO2 (red) and PEBP1 (green). DAPI (blue); *n* = 3 per group; Scale bar = 50 and 12.5 μm (enlarged). (B) Co‐IP images of PEBP1 and 15LO2; *n* = 3 per group. (C) Western blot images and quantitative analysis of phosphorylation of PEBP1 (*n* = 5 per group). Values are presented as mean ± SD. ****p < 0.0001 vs. the Ctrl group. 15LO2, 15‐lipoxygenase 2; DAPI, 4′,6‐diamidino‐2‐phenylindole; PEBP1, phosphatidylethanolamine binding protein 1.

As shown in Figure 3A, immunofluorescence indicated a stronger co‐localization of 15LO2 and PEBP1 after sevoflurane exposures, suggesting that enhanced interaction of 15LO2 and PEBP1 may exist. In addition, Co‐IP demonstrated that the direct interaction of 15LO2‐PEBP1 was enhanced by sevoflurane (Figure 3B). Furthermore, PEBP1 phosphorylation at Ser153 is elevated by sevoflurane (Figure 3C).

### 3.4 PD146176 mitigated sevoflurane‐induced ferroptosis and subsequent cognitive impairment

**FIGURE 4:**
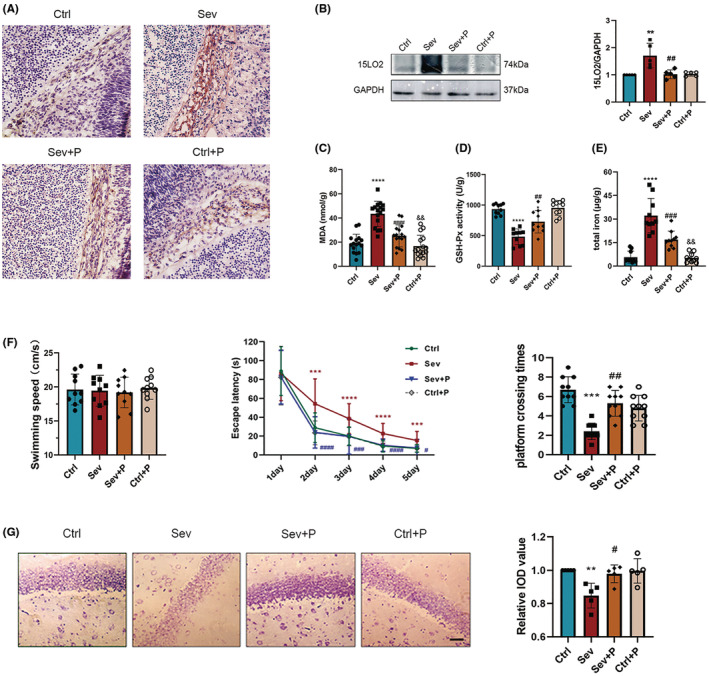
Effects of PD146176 on ferroptosis‐induced neurotoxicity in the offspring after maternal sevoflurane exposures. (A) Immunohistochemistry images of 15LO2 in the hippocampal rudiment in coronal sections; *n* = 5 per group; Scale bar = 100 μm. (B) Western blot bands and quantitative analysis of 15LO2 (*n* = 5 per group). (C) MDA levels, (D) Iron content, and (E) GPX4 activities in the fetal rat brain (*n* = 5 per group). (F) Results of MWM tests of the offspring from P28 to P33 (*n* = 10 per group): swimming speed, escape latencies and platform crossing times. (G) Images and quantitative analysis of Nissl's staining in P33 offspring (*n* = 5 per group). Values are presented as mean ± SD. **p < 0.01, ***p < 0.001 and ****p < 0.0001 vs. the Ctrl group; ^##^ *p* < 0.01, ^###^ *p* < 0.001 and ^####^ *p* < 0.0001 vs. the Sev group. ^&&^ *p* < 0.01 vs. the Sev + P group. 15LO2, 15‐lipoxygenase 2; GPX4, glutathione peroxidase 4; MDA, malondialdehyde; MWM, Morris water maze.

IHC and Western blotting results showed that PD146176 could downregulate the elevated expression of 15LO2 induced by sevoflurane (Figure 4A,B). PD146176 also reduced elevated MDA levels (Figure 4C) and significantly reduced iron overload (Figure 4D) after sevoflurane exposure. Meanwhile, PD146176 alleviated inhibition of GPX4 activity attributed to sevoflurane (Figure 4E). The offspring of pregnant rats in the Sev + P group had a shorter escape latency and more platform crossing times (Figure 4F). Additionally, pretreatment with PD146176 improved neural density and alignment of the CA1 region of the hippocampus during long‐term development (Figure 4G). These results suggest that inhibition of 15LO2 attenuates spatial learning memory deficits in offspring due to mid‐trimester sevoflurane exposures by inhibiting the ferroptosis process.

### 3.5 Sevoflurane mediates ferroptosis‐induced neurotoxicity via ATM and P53/SAT1 pathway

**FIGURE 5:**
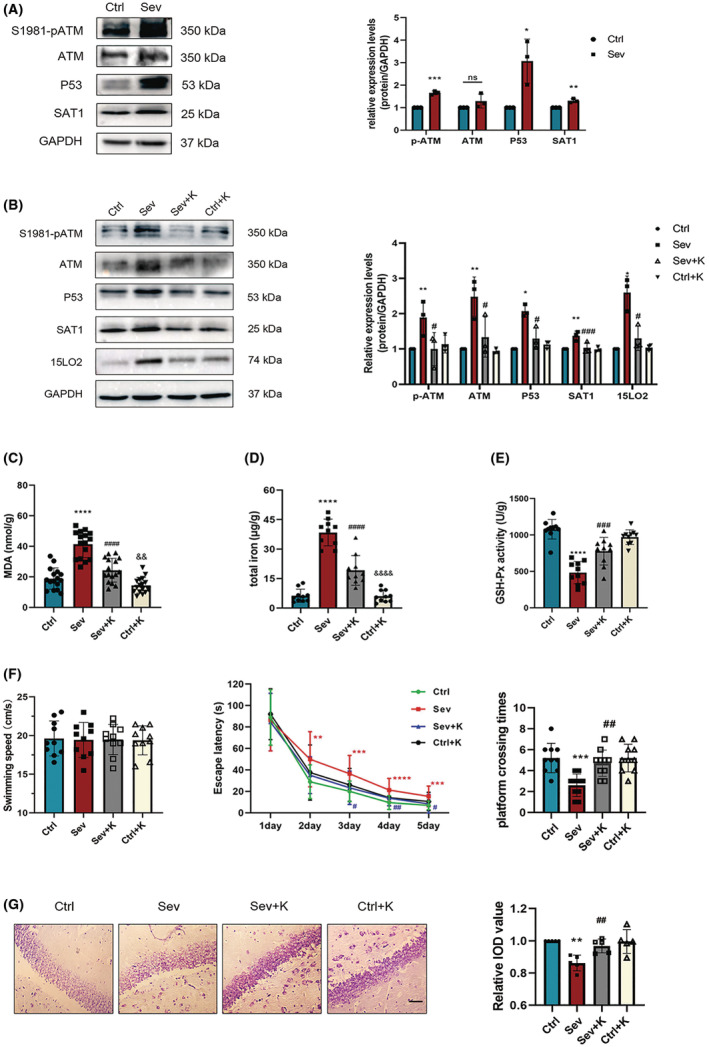
Effects of Ku55933 on ferroptosis‐induced neurotoxicity in the offspring after maternal sevoflurane exposures. (A) Western blot images and quantitative analysis of p‐ATM, ATM, P53 and SAT1 in the fetal rat brain of each group (*n* = 5 per group). (B) Expressions of p‐ATM, ATM, P53, SAT1 and 15LO2 after Ku55933: western blot images and quantitative analysis (*n* = 5 per group). (C) MDA levels, (D) Iron content, and (E) GPX4 activities in the fetal rat brain (*n* = 5 per group). (F) Results of MWM tests of the offspring from P28 to P33 (*n* = 10 per group): swimming speed, escape latencies and platform crossing times. (G) Images and quantitative analysis of Nissl's staining in P33 offspring (*n* = 5 per group). Values are presented as mean ± SD. **p < 0.01, ***p < 0.001 and ****p < 0.0001 vs. the Ctrl group; ^##^ *p* < 0.01, ^###^ *p* < 0.001 and ^####^ *p* < 0.0001 vs. the Sev group. ^&&^ *p* < 0.01 and ^&&&^ *p* < 0.001 vs. the Sev + K group. 15LO2, 15‐lipoxygenase 2; ATM, ataxia telangiectasia mutated; MWM, Morris water maze; SAT1, spermidine/spermine N 1‐acetyltransferase 1.

Given that 15‐lipoxygenase may act as the downstream effector of P53 and SAT1,^44^ and ATM may modulate P53‐induced ferroptosis,^41^ we hypothesized that ATM and P53/SAT1 pathway might participate in sevoflurane‐induced ferroptosis. ATM inhibitor Ku55933 was administered to further confirm our hypothesis. Herein, as well as elevated ATM and S1981 phospho‐ATM levels, sevoflurane had upregulated both P53 and SAT1 in fetal brains (Figure 5A). Nevertheless, these up‐regulations were reduced by Ku55933, including 15LO2 (Figure 5B). The results showed that sevoflurane‐induced MDA accumulation was significantly diminished by Ku55933 (Figure 5C). Meanwhile, Ku55933 limited iron overload and rescued the GPX4 activity suppression due to sevoflurane (Figure 5D,E). In addition, sevoflurane‐induced learning and memory impairment were restored by ATM inhibitors, as shown by shorter escape latencies, more platform‐crossing numbers (Figure 5F), and higher neural density (Figure 5G). In summary, we propose that 15LO2‐mediated neurotoxicity induced by sevoflurane may be associated with ATM activation and the P53/SAT1 signaling pathway.

### 3.6 Ku55933 inhibited the p‐ATM nuclear translocation in fetal rat brain induced by sevoflurane

**FIGURE 6:**
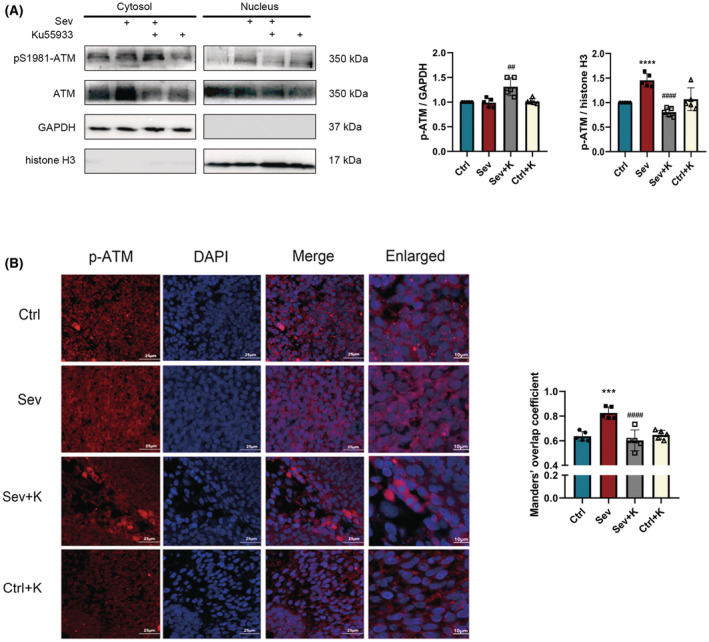
(A) Western blot images and quantitative analysis of p‐ATM and ATM in both plasma and nucleus of each group (*n* = 5 per group). (B) Immunofluorescence images and Manders' overlap coefficient of p‐ATM (red) and DAPI (blue). *n* = 3 per group; Scale bar = 25 and 10 μm (enlarged). Values are presented as mean ± SD. ***p < 0.001 and ****p < 0.0001 vs. the Ctrl group; ^##^ *p* < 0.01 and ^####^ *p* < 0.0001 vs. the Sev group. ATM, ataxia telangiectasia mutated; DAPI, 4′,6‐diamidino‐2‐phenylindole.

As shown in Figure 6A, ATM and S1981 phospho‐ATM were observed to be enriched in the nucleus after exposure to sevoflurane. Interestingly, Ku‐55933 reduced ATM phosphorylation at S1981 in the nucleus. Consistently, immunofluorescence also confirmed that the effects of sevoflurane on p‐ATM nuclear translocation were eliminated by Ku55933 (Figure 6B).

## 4 DISCUSSION

This study revealed that ferroptosis contributed to neurotoxicity induced by multiple sevoflurane exposures during the mid‐trimester in offspring, and 15LO2‐PEBP1 was one of the major triggers for sevoflurane‐induced ferroptosis in fetal brains presumably via a mechanism via ATM activation and its downstream P53/SAT1 signaling. Ferroptosis inhibition or inhibition of 15LO2 or ATM protects fetal rats from neurotoxicity induced by maternal sevoflurane exposures.

Nowadays, emerging evidence has shown that maternal sevoflurane exposure during pregnancy may induce neurotoxic effects in the fetus, resulting in impaired neurodevelopment in offspring.^4^, ^9^, ^47^, ^49^, ^50^ Sevoflurane is the preferred inhalation anesthetic during pregnancy and has many benefits, such as rapid induction of anesthesia and quicker awakening,^3^ and modulation of its depth of anesthesia depends mainly on changing concentration. Sevoflurane at 3.0% is equivalent to a clinical concentration of 1.3–1.5 minimum alveolar concentration (MAC), which is close to the concentration commonly used in clinical practice during pregnancy and neonatal surgery. Other researchers have also investigated the effects of single 6‐hour versus triple 2‐hour sevoflurane exposures on the rodent brain, and the results suggest that repeated sevoflurane exposures are more likely to produce negative outcomes in the rodent brain.^3^ Similarly, in this study, we found that maternal exposure to 3.0% sevoflurane for 3 consecutive days during mid‐pregnancy reduced neurons in the neonatal hippocampus, along with long‐term neuronal density reduction in the hippocampus and impaired learning memory function in offspring, which may be strongly related to sevoflurane‐induced neuronal ferroptosis in the fetal brain.

Meanwhile, ferroptosis is a novel form of programmed cell death characterized by intracellular iron‐dependent accumulation of lipid peroxidation,^13^ it has been revealed to be extensively involved in the onset and development of neurological disorders, particularly those associated with reactive oxygen species (ROS).^51^, ^52^, ^53^ The developing brain is characterized by higher oxygen consumption, high levels of unsaturated fatty acid, and poor antioxidant levels, making it more vulnerable to oxidative stress.^54^

In this paper, midgestational rats exposed to 3.0% sevoflurane for 3 consecutive days triggered lipid peroxidation and iron accumulation in the fetal rat brain, leading to ferroptosis in neural cells, resulting in a reduction of hippocampal neurons and learning memory deficits in offspring. At the molecular level, sevoflurane activated ATM and its downstream P53/SAT1 pathway, thereby upregulating 15LO2 expression and enhancing 15LO2‐PEBP1 interaction in fetal brains. These results suggest that ferroptosis mediated by 15LO2‐PEBP1 and ATM activation may contribute to the mechanisms of sevoflurane neurotoxicity. In addition, Fer‐1, PD146176, or Ku55933 rescued sevoflurane‐induced ferroptosis and learning and memory deficits, indicating that specific inhibition of ferroptosis and targets may have beneficial effects on sevoflurane‐induced neurotoxicity.

In most ferroptosis events, lipid peroxidation enzyme expression (eg, ACSL4) is upregulated, while GPX4 expression is downregulated or its activity is impaired. Inhibition of expression or activity of SLC7A11 and GPX4 will lead to ROS accumulation and ferroptosis.^11^ Our study revealed that after maternal multiple sevoflurane exposures, ferroptosis onset in the fetal brain. Evidence that elevated expression of ACSL4 and PTGS2 as well as decreased expression of SLC7A11 due to sevoflurane and the presence of shrunken mitochondria in neuronal cells confirmed the onset of ferroptosis; whereas GPX4 expression was not significantly altered in our study, but its enzyme activity was inhibited, again supporting the findings. Furthermore, in the process of ferroptosis, another critical component of ferroptosis is intracellular iron overload. When excess Fe^2+^ is generated intracellularly, it can trigger ferroptosis by catalyzing the generation and massive accumulation of lipid ROS in the cell through the Fenton reaction.^55^ As a major substrate of ferritinophagy,^56^ FTH1 decreased and iron content increased, indicating that enhanced ferritinophagy may occur in ferroptosis induced by sevoflurane.

15‐Hydroperoxy‐eicosatetraenoyl‐phosphatidylethanolamine (15‐HpETE‐PE) is produced by the 15LOX‐PEBP1 complex or by an iron‐catalyzed nonenzymatic radical reaction and acts as a ferroptosis signal.^35^ In ferroptosis driven by the discordance of iron and lipids, the lipid peroxidation product 15‐HpETE‐PE accumulates and carries out the mechanisms of injury.^57^ Fer‐1 has been shown to have a poor inhibitory effect on 15LOX but counteracts the role of 15LOX in ferroptosis by binding to the 15LOX/PEBP1 complex and disrupting its catalysis‐dependent allosteric motions, in terms of reducing HpETE‐PE production.^57^ Herein, sevoflurane enhanced the 15LO2‐PEBP1 interaction, provoking a prominent increase in lipid peroxidation levels in the fetal rat brain, which triggered ferroptosis. PEBP1 has been reported to be involved in neuronal death and inflammation following cerebral ischemia‐reperfusion injury.^58^, ^59^ Phosphorylation at the Ser153 site of PEBP1 serves as a functional switch for PEBP1 by mechanism, presumably because phosphorylation at the Ser153 site affects the interactions of PEBP1 with other molecules.^58^, ^60^ Considering that PEBP1 functions as a rheostat for regulating ferroptosis, autophagy, and maintaining cell integrity,^61^, ^62^ we hypothesized that PEBP1 may function a crucial role in the crosstalk between ferroptosis and other phenotypes, such as autophagy.

Erastin is a ferroptosis inducer that directly inhibits the activity of SLC7A11 with the ROS‐dependent and iron‐dependent mechanism.^10^ Interestingly, erastin‐induced ferroptosis could be rescued by LOX inhibitors, suggesting that the LOXs‐mediated ferroptosis mechanism may also be related to SLC7A11 inhibition.^12^ Combined with our study, sevoflurane exposure downregulated SLC7A11 and upregulated 15LO2 expression in fetal rat brains, as well as diminished GPX4 activity. Besides, inhibition of 15LO2 could somewhat alleviate cognitive impairment in offspring due to sevoflurane. Therefore, we speculate that ferroptosis induced by sevoflurane in neuronal cells might also be attributed to lipid peroxidation mediated by 15LO2 in the inhibited state of SLC7A11 and GPX4.

ATM can be activated by two principal pathways^63^, ^64^, ^65^: the classical ATM activation pathway depends on the DNA damage response; the other pathway involves formatting disulfide bonds between ATM monomers under oxidative stress conditions. ATM activation is also associated with mitochondrial dysfunction^66^, ^67^ and calcium homeostasis imbalance.^68^ Notably, it has been published that sevoflurane, isoflurane, and desflurane can induce DNA damage^69^, ^70^ and generate excessive oxidative stress.^71^ ATM itself can protect cells from double‐stranded DNA damage by activating P53 and its regulators murine double minute X (MDMX) and MDM2.^72^ Regulation of DNA excision repair activities has been identified as associated with nuclear translocation. Additionally, the nuclear translocation of certain proteins depends on their phosphorylation state to the same extent. Moreover, *SAT1* gene is a transcription target of P53, which can induce vulnerability to ferroptosis on ROS.^45^ Moreover, *SAT1* gene is also associated with ALOX15 expression, and SAT1‐induced ferroptosis could be significantly abrogated by PD146176.^44^ Our findings revealed that sevoflurane activates ATM and promotes p‐ATM nuclear translocation, ultimately upregulating downstream P53/SAT1/15LO2. Taken together, we hypothesize that the mechanism by which ATM mediates ferroptosis through regulation of P53/SAT1/15LO2 may be correlated with enhanced nuclear translocation.

In this study, we establish that ferroptosis might contribute to sevoflurane‐induced neurotoxicity in the developing brain. However, the proportional contribution of other phenotypes should be noted as sevoflurane‐induced intracellular stress and ROS accumulation may also affect other pathways. Although the interplay between ferroptosis and neurotoxicity of anesthetics on the developing brain requires further investigation, our findings provide promising insights into mitigating the neurotoxic effects of sevoflurane on the developing brain.

There are certain limitations to be noted. Firstly, the sensitivity of individuals to the toxicity of anesthetics is variable. However, in the present study, no distinction was made between susceptible and non‐susceptible cases. For some individuals with variability in vulnerability, individual variability and underlying mechanisms warrant further investigation. Secondly, due to experimental conditions, the level of 15‐HpETE‐PE could not be detected temporarily. The above deficiencies need to be further studied. Finally, in terms of the time point chosen for sampling after sevoflurane exposures, only one time point was reported in this paper (ie 12 h after exposure) because given the selection of 3, 6, 24, and 48 h in our earlier pre‐experiments, we found that 12 h was the most typical time point for most index measurements. With this in mind, it is worth further investigating whether the process of sevoflurane‐induced ferroptosis in neuronal cells is time‐dependent, or whether damage or repair mechanisms persist.

## 5 CONCLUSIONS

Finally, this study confirms the contribution of ferroptosis to neurotoxicity induced by maternal sevoflurane anesthesia during the mid‐trimester in offspring and its mechanism might be attributed to upregulation of 15LO2 via ATM/P53/SAT1 pathway and enhancement of 15LO2‐PEBP1 interaction. Furthermore, these findings indicated that inhibition of ferroptosis may become a potential approach to ameliorate the neurotoxic effects of sevoflurane.

## AUTHOR CONTRIBUTIONS

Conceptualization and methodological work were performed by Ping Zhao and Qian Jiang; data curation was conducted by Qian Jiang and Qiushi Gao; manuscript was written by Qian Jiang; visualization, supervision, and verification were performed by Qian Jiang and Cong Wang; manuscript was revised by Ping Zhao and Ziyi Wu; all authors read and approved the final manuscript.

## CONFLICT OF INTEREST STATEMENT

The authors declare no conflict of interests.

## Supporting information


Supplementary material

## Data Availability

The data that support the findings of this study are available from the corresponding author upon reasonable request.
