# Correction to “Multiple Sevoflurane Exposures During Mid‐Trimester Induce Neurotoxicity in the Developing Brain Initiated by 15LO2‐Mediated Ferroptosis”

**DOI:** 10.1002/cns.70685

**Published:** 2025-12-19

Q. Jiang, C. Wang, Q. Gao, et al., “Multiple Sevoflurane Exposures During Mid‐Trimester Induce Neurotoxicity in the Developing Brain Initiated by 15LO2‐Mediated Ferroptosis,” *CNS Neuroscience & Therapeutics* 29, no. 10(2023): 2972–2985. https://doi.org/10.1111/cns.14236.

In the original version of this article, there was an error in Figure 2G and Figure 4G. Specifically, the immunohistochemical image of NeuN for the Sev+F group in Figure 2G and Nissl's staining image for the “Ctrl” group in Figure 4G were incorrect. Additionally, in Figure 5G, it showed the wrong Nissl's staining image in “Ctrl+K” group. The correct images are provided below. The corrections do not change the results or conclusions of this paper.

**FIGURE 2:**
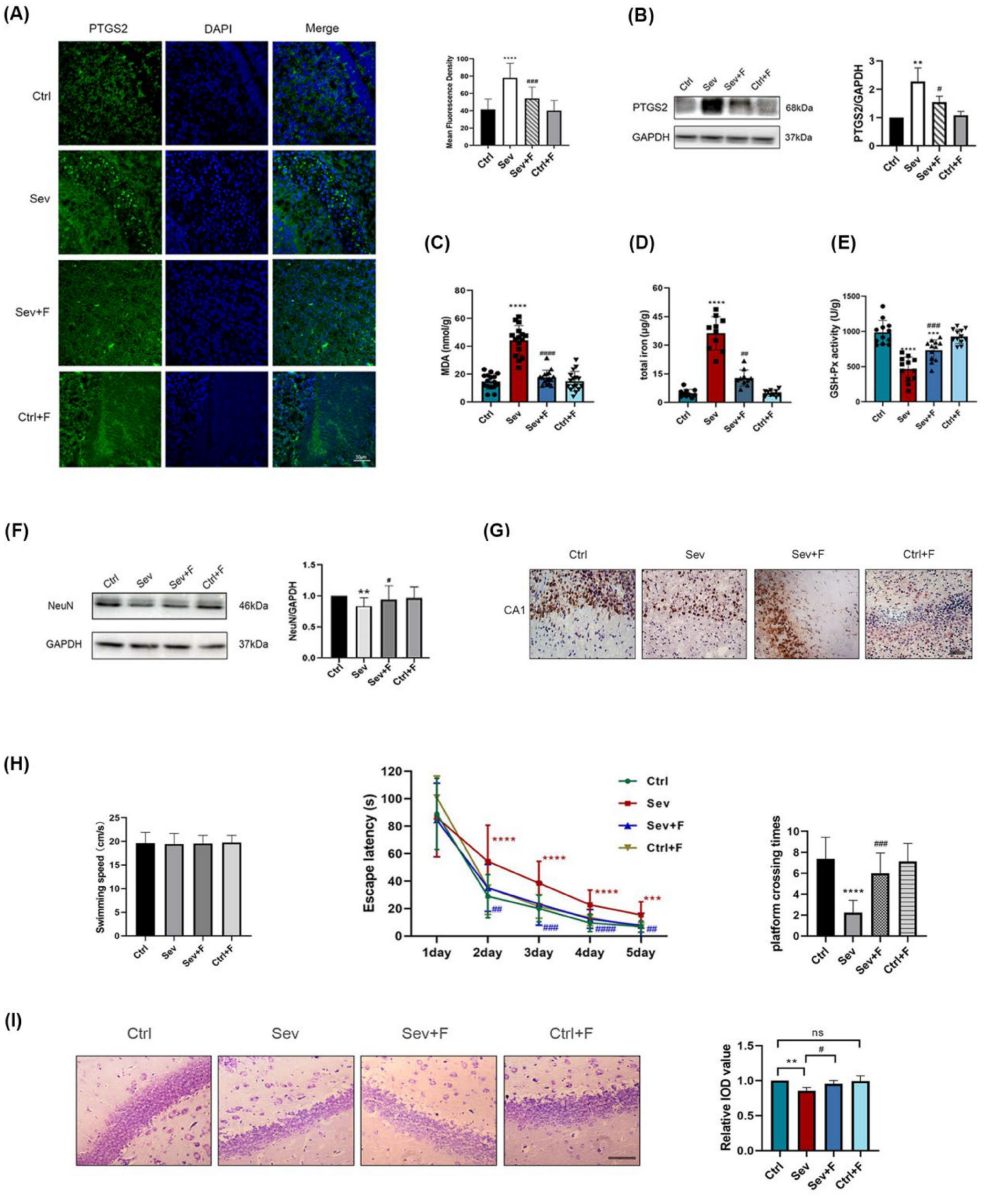
Effects of Fer‐1 on ferroptosis‐induced neurotoxicity in the offspring after maternal sevoflurane exposures.

**FIGURE 4:**
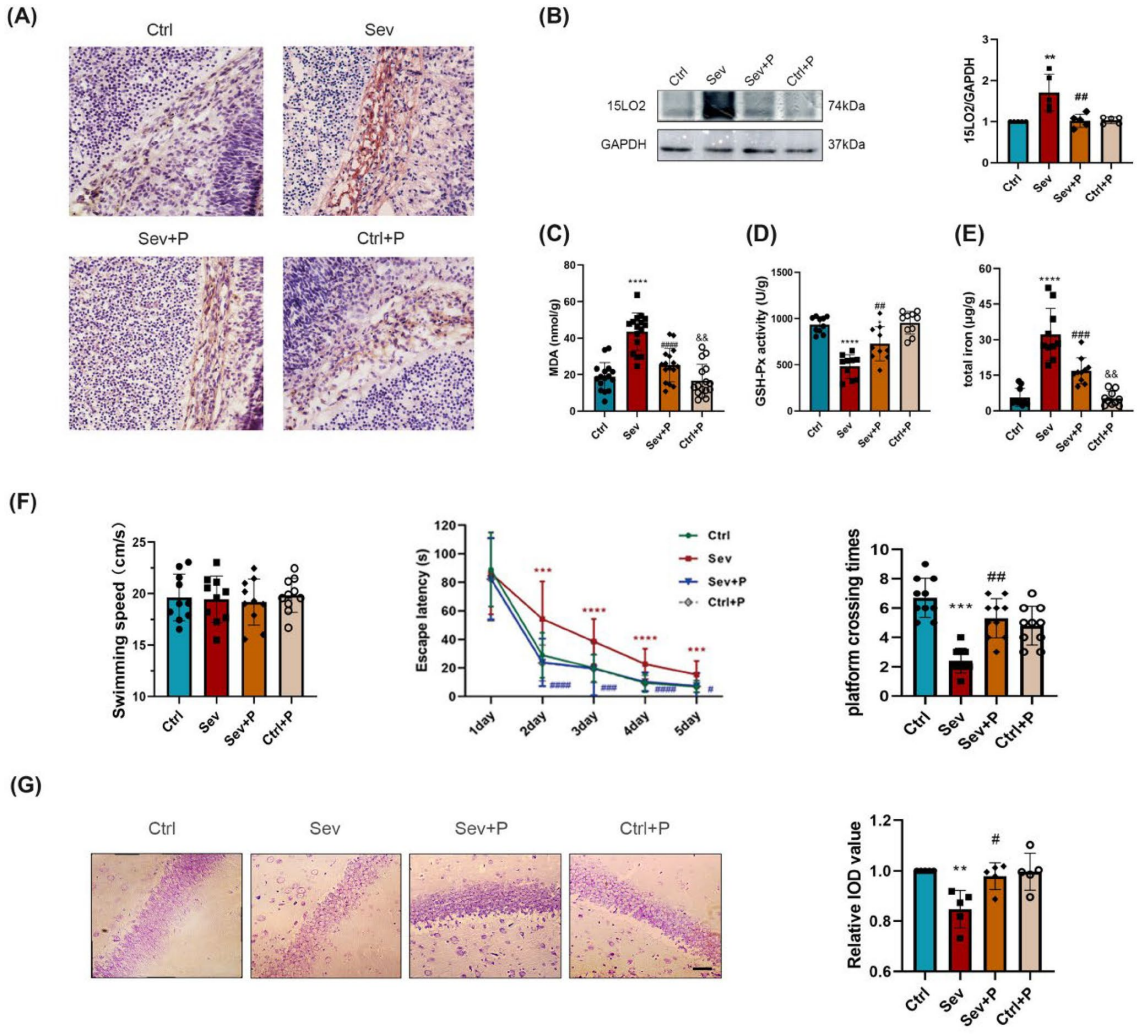
Effects of PD146176 on ferroptosis‐induced neurotoxicity in the offspring after maternal sevoflurane exposures.

**FIGURE 5:**
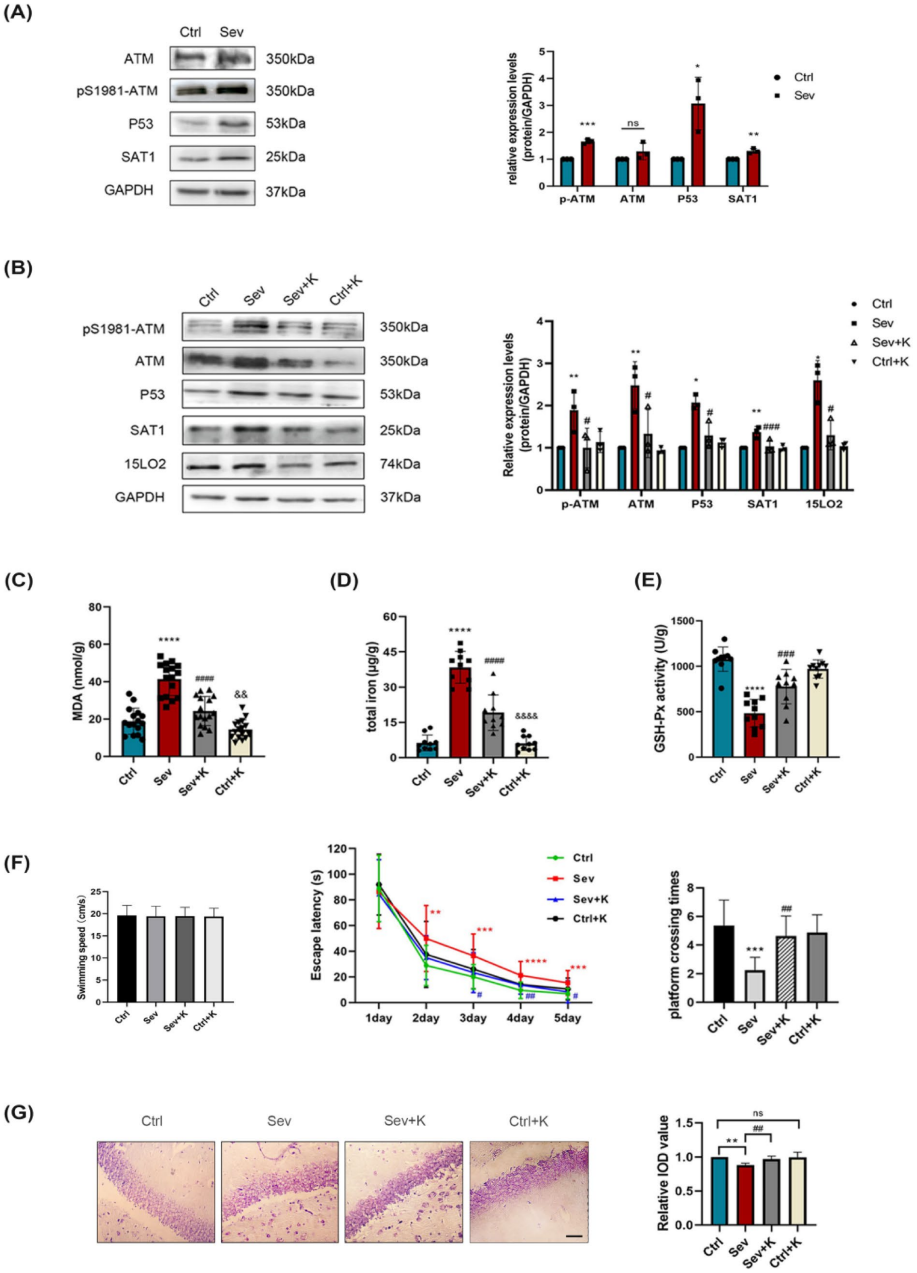
Effects of Ku55933 on ferroptosis‐induced neurotoxicity in the offspring after maternal sevoflurane exposures.

We apologize for this error.

