# Supplementary material for: Multiple sevoflurane exposures during mid‐trimester induce neurotoxicity in the developing brain initiated by 15LO2‐Mediated ferroptosis
Source: CNS Neurosci Ther. 2023 Jun 7;29(10):2972–85. doi: 10.1111/cns.14236 (PMC10493671; doi:10.1111/cns.14236)

Manuscript ID: CNSNT-2023-178

Multiple Sevoflurane Exposures during Mid-trimester Induces Neurotoxicity in the Developing Brain Initiated by 15LO2-Mediated Ferroptosis

Qian Jiang, Cong Wang, Qiushi Gao, Ziyi Wu, Ping Zhao

Figure 1

Full unedited gels/blots for Figure 1B

GPX4 (22 kDa)

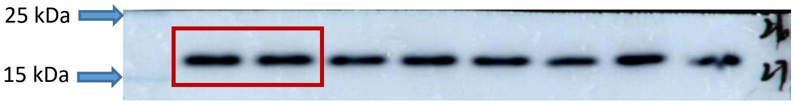

--Image of GAPDH for GPX4:

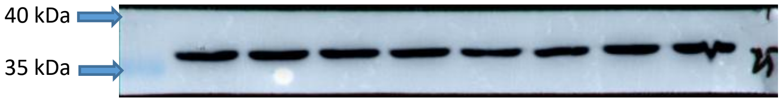

ACSL4 (79 kDa)

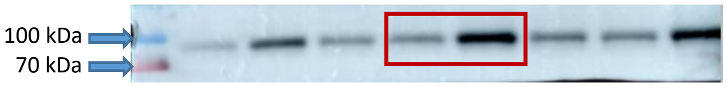

--Image of GAPDH for ACSL4:

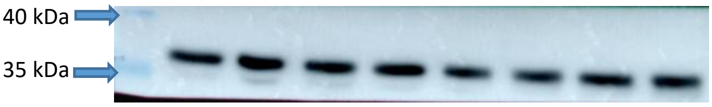

15LO2 (74 kDa)

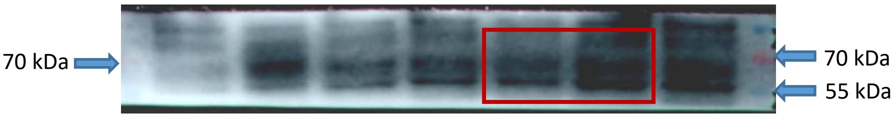

SLC7A11 (55 kDa)

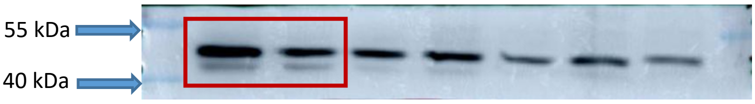

FTH1 (21 kDa)#

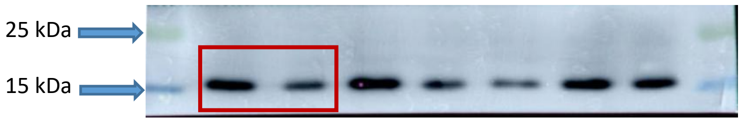

GAPDH (37 kDa)

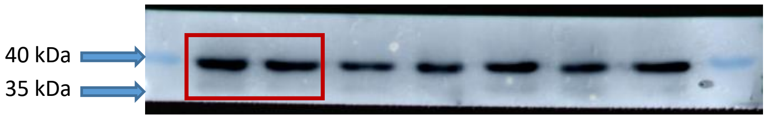

Figure 2

Full unedited gels/blots for Figure 2B

PTGS2 (68 kDa)

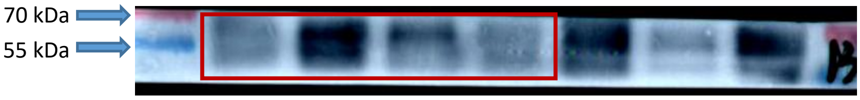

GAPDH (37 kDa)

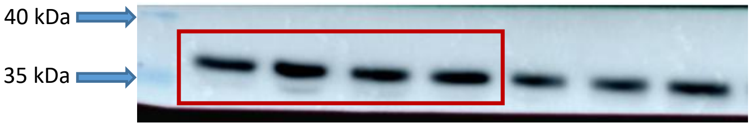

Full unedited gel/blot for Figure 2F

NeuN (46 kDa)

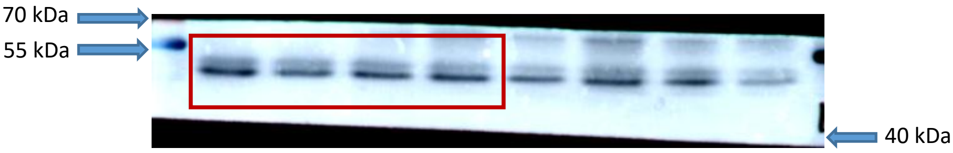

GAPDH (37 kDa)

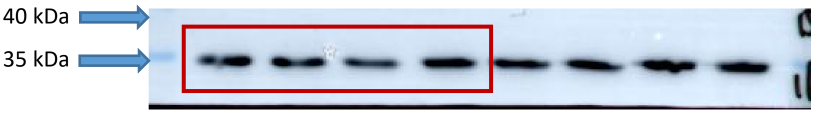

Figure 3

Full unedited gel/blot for Figure 3B

Input:

15LO2 (74 kDa)

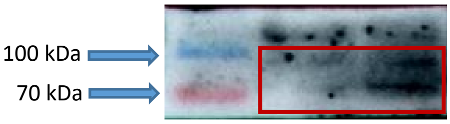

---Image of GAPDH for 15LO2

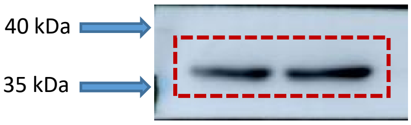

PEBP1 (21 kDa)

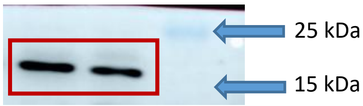

GAPDH (37 kDa)

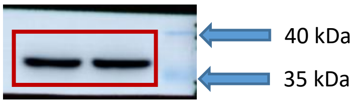

IP:

15LO2-15LO2

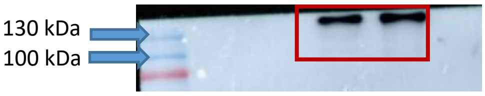

PEBP1-PEBP1

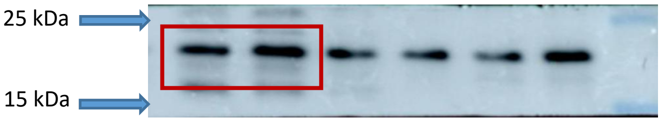

15LO2-PEBP1

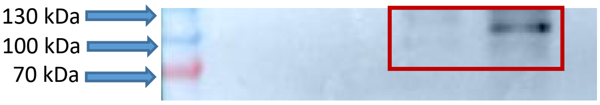

PEBP1-15LO2: .

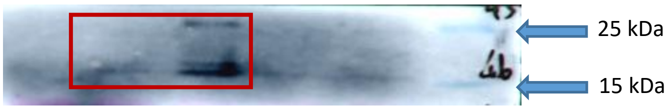

Full unedited gel/blot for Figure 3C

p-PEBP1 (21 kDa)

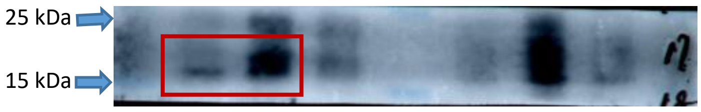

--Image of GAPDH for p-PEBP1:

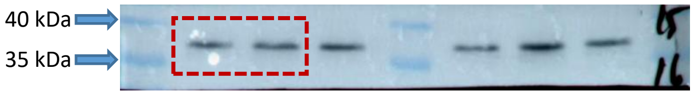

PEBP1 (21 kDa)

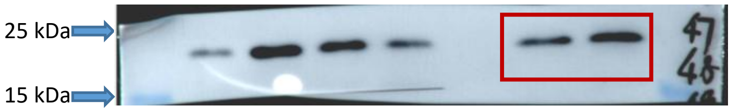

GAPDH (37 kDa)

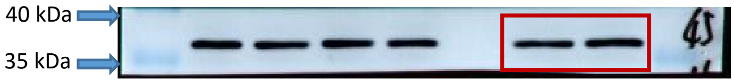

Figure 4

Full unedited gel/blot for Figure 4B

15LO2 (74 kDa)

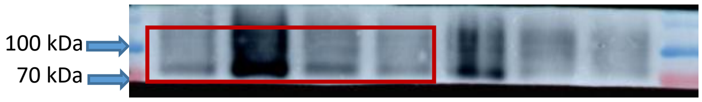

GAPDH (37 kDa)

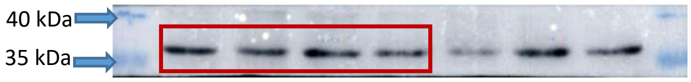

Figure 5

Full unedited gel/blot for Figure 5A

p-ATM (350 kDa)#

ATM (350 kDa)#

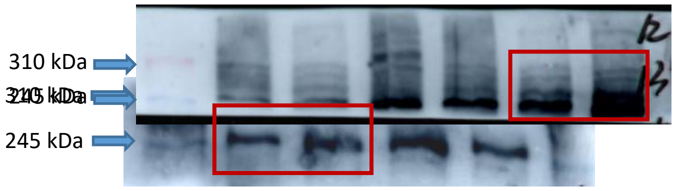

P53 (53 kDa)

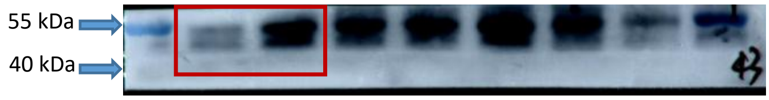

SAT1 (25 kDa)

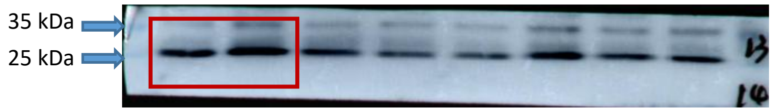

GAPDH (37 kDa)

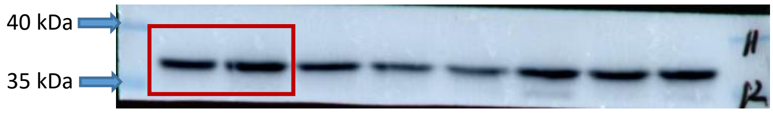

Full unedited gel/blot for Figure 5B

p-ATM (350 kDa)#

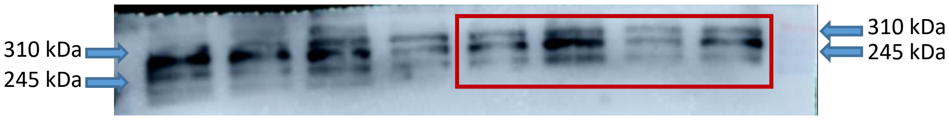

ATM (350 kDa)#

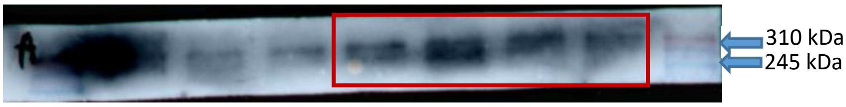

P53 (53 kDa)

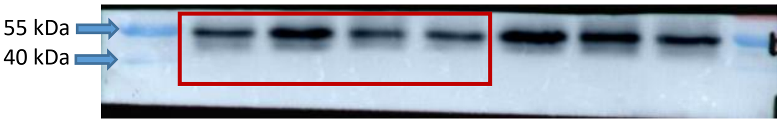

SAT1 (25 kDa)

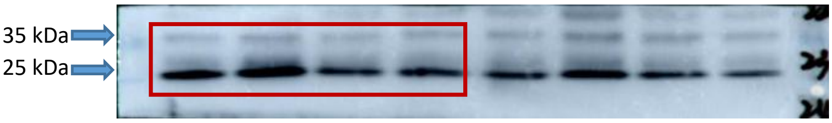

15LO2 (74 kDa)

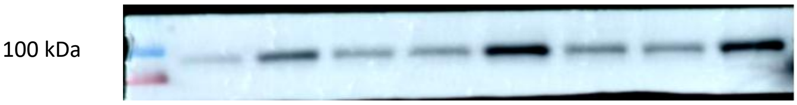

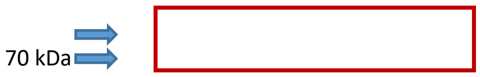

GAPDH (37 kDa)

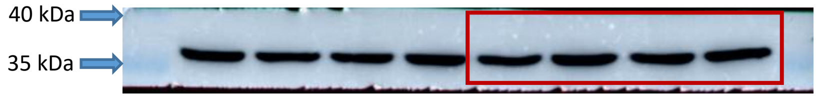

Figure 6

Full unedited gel/blot for Figure 6A

-cytosol#

p-ATM (350 kDa)

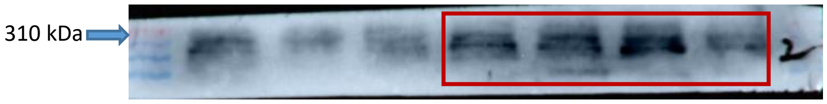

ATM (350 kDa)

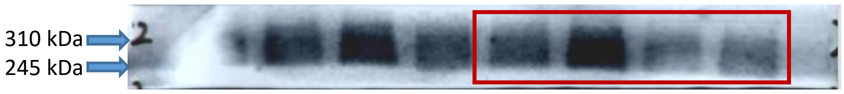

GAPDH (37 kDa)

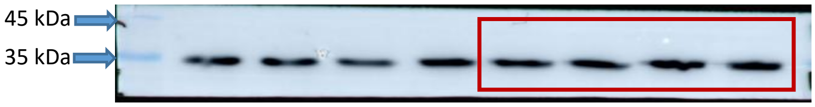

Histone H3 (17 kDa)

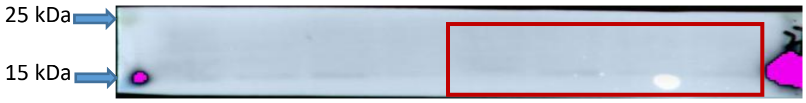

-nucleus#

p-ATM (350 kDa)

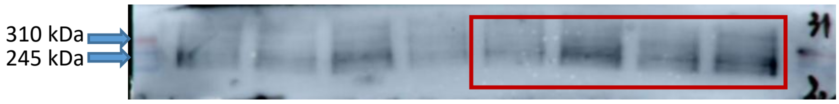

ATM (350 kDa)

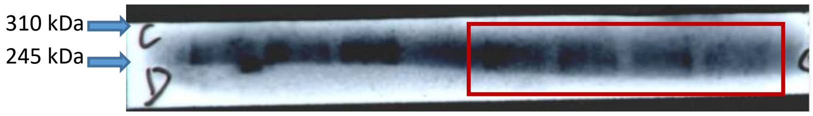

GAPDH (37 kDa)

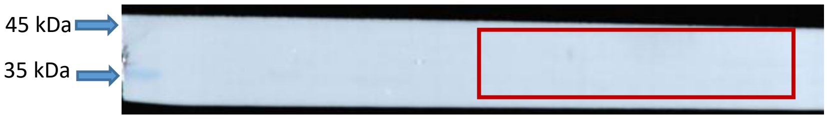

Histone H3 (17 kDa)

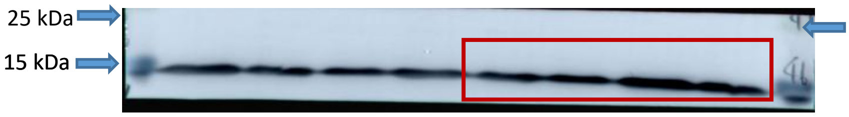

#These bands (gels) were labeled with Extra Range Prestained Protein Marker (PL00003, proteintech, USA), and the corresponding molecular weight is shown in the following supplementary image at the end of this file.

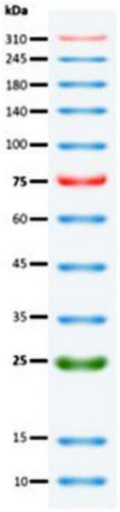

Supplement: Supplementary file 1 — Supplementary material [file CNS-29-2972-s001.pdf]
